# α_2_-Adrenergic Disruption of β Cell BDNF-TrkB Receptor Tyrosine Kinase Signaling

**DOI:** 10.3389/fcell.2020.576396

**Published:** 2020-10-15

**Authors:** Michael A. Kalwat, Zhimin Huang, Derk D. Binns, Kathleen McGlynn, Melanie H. Cobb

**Affiliations:** Department of Pharmacology, UT Southwestern Medical Center, Dallas, TX, United States

**Keywords:** cell signaling, pancreatic islet, extracellular-signal-regulated kinase, brain-derived neurotrophic factor, BDNF/NT-3 growth factors receptor, epinephrine, adrenergic receptor, diabetes

## Abstract

Adrenergic signaling is a well-known input into pancreatic islet function. Specifically, the insulin-secreting islet β cell expresses the G_i/o_-linked α_2_-adrenergic receptor, which upon activation suppresses insulin secretion. The use of the adrenergic agonist epinephrine at micromolar doses may have supraphysiological effects. We found that pretreating β cells with micromolar concentrations of epinephrine differentially inhibited activation of receptor tyrosine kinases. We chose TrkB as an example because of its relative sensitivity to the effects of epinephrine and due to its potential regulatory role in the β cell. Our characterization of brain-derived neurotrophic factor (BDNF)-TrkB signaling in MIN6 β cells showed that TrkB is activated by BDNF as expected, leading to canonical TrkB autophosphorylation and subsequent downstream signaling, as well as chronic effects on β cell growth. Micromolar, but not nanomolar, concentrations of epinephrine blocked BDNF-induced TrkB autophosphorylation and downstream mitogen-activated protein kinase pathway activation, suggesting an inhibitory phenomenon at the receptor level. We determined epinephrine-mediated inhibition of TrkB activation to be G_i/o_-dependent using pertussis toxin, arguing against an off-target effect of high-dose epinephrine. Published data suggested that inhibition of potassium channels or phosphoinositide-3-kinase signaling may abrogate the negative effects of epinephrine; however, these did not rescue TrkB signaling in our experiments. Taken together, these results show that (1) TrkB kinase signaling occurs in β cells and (2) use of epinephrine in studies of insulin secretion requires careful consideration of concentration-dependent effects. BDNF-TrkB signaling in β cells may underlie pro-survival or growth signaling and warrants further study.

## Introduction

Glucose homeostasis is largely controlled by the metered secretion of insulin from pancreatic islet β cells. β cells respond to elevated circulating glucose via coupling its metabolism to membrane depolarization, calcium (Ca^2+^) influx, and insulin exocytosis (Kalwat and Cobb, 2017). Secreted insulin suppresses liver gluconeogenesis and stimulates peripheral glucose uptake. Diabetes is a disease of hyperglycemia caused by deficient insulin production and action. In diabetes, β cells are either destroyed by the immune system (type 1 diabetes) or unable to secrete sufficient insulin in response to stimulation (type 2 diabetes). In order to function properly, pancreatic β cells integrate a diverse array of inputs, including nutrients and hormones. To accomplish this, β cells utilize a variety of signaling mechanisms such as G-protein-coupled receptors (GPCRs) (Holst, 2007; Straub and Sharp, 2012) and receptor tyrosine kinases (RTKs) (Kulkarni et al., 1999, 2002; Song et al., 2016). Reported cross talk between GLP1R and EGFR in islet β cells lends support to the idea of more general GPCR-RTK signaling interactions in β cells (Fusco et al., 2017).

Extracellular regulated kinase 1/2 (ERK1/2) is activated by insulin secretagogues (e.g., glucose, amino acids) and blunted by inhibitors of secretion (e.g., epinephrine) and is therefore frequently used as a proxy for β cell responsiveness (Longuet et al., 2005; Jaques et al., 2008; Goehring et al., 2011). ERK1/2 activation has long been recognized for its role in β cell growth and insulin gene expression (Hugl et al., 1998; Briaud et al., 2003; Khoo et al., 2003; Lawrence et al., 2008). Recently, acute ERK2 activity was demonstrated to be critical for the first phase of insulin secretion (Leduc et al., 2017). Our interest in the pathways leading to ERK1/2 activation and the inhibitory functions of epinephrine in β cells led us to test the impact of epinephrine on RTK signaling to ERK1/2. Epinephrine has different effects on the ERK1/2 pathway depending on cell type and receptors expressed. In β cells, epinephrine activates α_2_-adrenergic receptors and inhibits insulin secretion as well as glucose-stimulated ERK1/2 activation (Peterhoff et al., 2003; Gibson et al., 2006).

We discovered that epinephrine suppressed RTK signaling in a concentration-dependent manner and with varying potency depending on the RTK. Activation of α_2_-adrenergic receptors in pancreatic islet β cells has been extensively studied and is well-known to suppress or completely inhibit insulin secretion through Gα_i/o_-dependent signaling (Sharp, 1996; Straub and Sharp, 2012). While physiological circulating concentrations of catechols (epinephrine, norepinephrine) range from picomolar to low nanomolar (Clutter et al., 1980; Dodt et al., 1997; Kienbaum et al., 1998), often micromolar concentrations are used to investigate pancreatic islet function (Sieg et al., 2004; Gibson et al., 2006; Iwanir and Reuveny, 2008; Zhao et al., 2008; Zhang et al., 2009; Slucca et al., 2010; Tian et al., 2011). Among the RTKs we tested in β cells, we chose TrkB for its sensitivity to stimulation with ligand, inhibition by epinephrine, and relative lack of knowledge of its role in β cells. Our characterization and analysis of BDNF-TrkB signaling to ERK1/2 in MIN6 β cells revealed effects on growth and interactions with insulin secretagogues and that epinephrine blocks TrkB signaling at the receptor level in a G_i_-dependent manner. We conclude from our findings that the doses of epinephrine used in β cell experiments should be carefully considered.

## Materials and Methods

### Antibodies, Plasmids, and Reagents

All chemicals were purchased through Fisher Scientific unless otherwise indicated and listed in Supplementary Table 1. All relevant reagents used in this study are listed in Supplementary Table 1. Concentrations of compounds and ligands were chosen based either on the literature or on empirical testing in MIN6 cells with dose–response curves. For BDNF, the 10-ng/mL dose was chosen based on dose–response curve stimulations of ERK1/2 activation in MIN6 cells. Above that dose, no substantial increase in pERK1/2 was observed. As the dose of epinephrine is a major point of this work, we used commonly used micromolar doses found in the literature, as well as less frequently used nanomolar doses in our experiments.

### Immunoblotting

Cleared cell lysates (40–50 μg) were separated on 10% gels by SDS-PAGE and transferred to nitrocellulose for immunoblotting. All membranes were blocked in Odyssey blocking buffer (Licor) for 1 h before overnight incubation with primary antibodies diluted in blocking buffer. After three 10-min washes in 20 mM Tris–HCl pH 7.6, 150 mM NaCl, 0.1% Tween-20 (TBS-T), membranes were incubated with fluorescent secondary antibodies for 1 h at room temperature. After three 10-min washes in TBS-T, membranes were imaged on a Licor Odyssey scanner.

### MIN6 Cell Culture and Transfections

MIN6 β cells were cultured in Dulbecco’s modified Eagle’s medium (D6429), supplemented with 15% fetal bovine serum, 100 units/ml penicillin, 100 μg/ml streptomycin, 292 μg/ml L-glutamine, and 50 μM β-mercaptoethanol (Kalwat et al., 2016). MIN6 cells in 12-well dishes were untreated or transfected with Lipofectamine 2000 according to the manufacturer’s instructions and cultured 48 h before use in experiments. For chronic BDNF treatment, cells were incubated with 100 ng/ml BDNF in complete culture media. Prior to stimulation, MIN6 cells were washed twice with and incubated for 2 h in freshly prepared glucose-free modified Krebs–Ringer bicarbonate buffer (MKRBB: 5 mM KCl, 120 mM NaCl, 15 mM HEPES, pH 7.4, 24 mM NaHCO_3_, 1 mM MgCl_2_, 2 mM CaCl_2_, and 1 mg/ml radioimmunoassay-grade BSA). Cells were lysed in 25 mM HEPES, pH 7.4, 1% Non-idet P-40, 10% glycerol, 50 mM sodium fluoride, 10 mM sodium pyrophosphate, 137 mM NaCl, 1 mM sodium vanadate, 1 mM phenylmethylsulfonyl fluoride, 10 μg/ml aprotinin, 1 μg/ml pepstatin, and 5 μg/ml leupeptin and cleared of insoluble material by centrifugation at 10,000 × *g* for 10 min at 4°C for subsequent use.

### Human Pancreatic Tissue Microscopy

Paraffin-embedded formalin-fixed 5-μm sections of de-identified human pancreas tissue on glass slides were obtained through the Simmons Comprehensive Cancer Center at UT Southwestern Medical. Slides were deparaffinized with the assistance of the UTSW Molecular Pathology Core using an automated system for xylene and ethanol washes. Antigen retrieval was performed by heating in citrate buffer^1^. After three 10-min washes in PBS-T (137 mM NaCl, 2.7 mM KCl, 10 mM Na_2_HPO_4_, 1.8 mM KH_2_PO_4_, pH 7.4, 0.05% Tween-20), slides were blocked for 1 h at room temperature in normal donkey serum (NDS) block solution (2% donkey serum, 1% bovine serum albumin, 0.1% cold fish skin gelatin, 0.1% Triton X-100, 0.05% sodium azide, PBS-T). Sections were outlined with a barrier pen and incubated overnight at 4°C with primary antibodies. Primary antibodies were diluted in NDS blocking solution at the indicated dilutions (Supplementary Table 1). After three 10-min washes in PBS-T, slides were incubated in secondary antibodies in NDS block for 1 h at room temperature. The washed slides were mounted with Dapi Fluoromount-G (SouthernBiotech #0100-20) and imaged on either an LSM700 Zeiss AxioObserver confocal microscope equipped with a Plan-Apochromat 20x/0.8 M27 objective and a MBS 405/488/555/639 beam splitter. Laser lines were 639 nm (for TrkB), 555 nm (for Insulin), 488 nm (for Glucagon), and 405 nm (for DAPI) each at 2% power. Images were processed in Zeiss’ Zen software to add scale bars, set coloration for channels, and generate merged images. Scale bars indicate 50 μm.

### Statistical Analysis

Quantitated data are expressed as mean ± SD. Data were evaluated using Student’s t test or ANOVA with multiple comparisons test as appropriate and considered significant if *P* < 0.05. Graphs were made in GraphPad Prism 8.

## Results

### Epinephrine Differentially Blocks Activation of RTK Signaling in MIN6 β Cells

**FIGURE 1:**
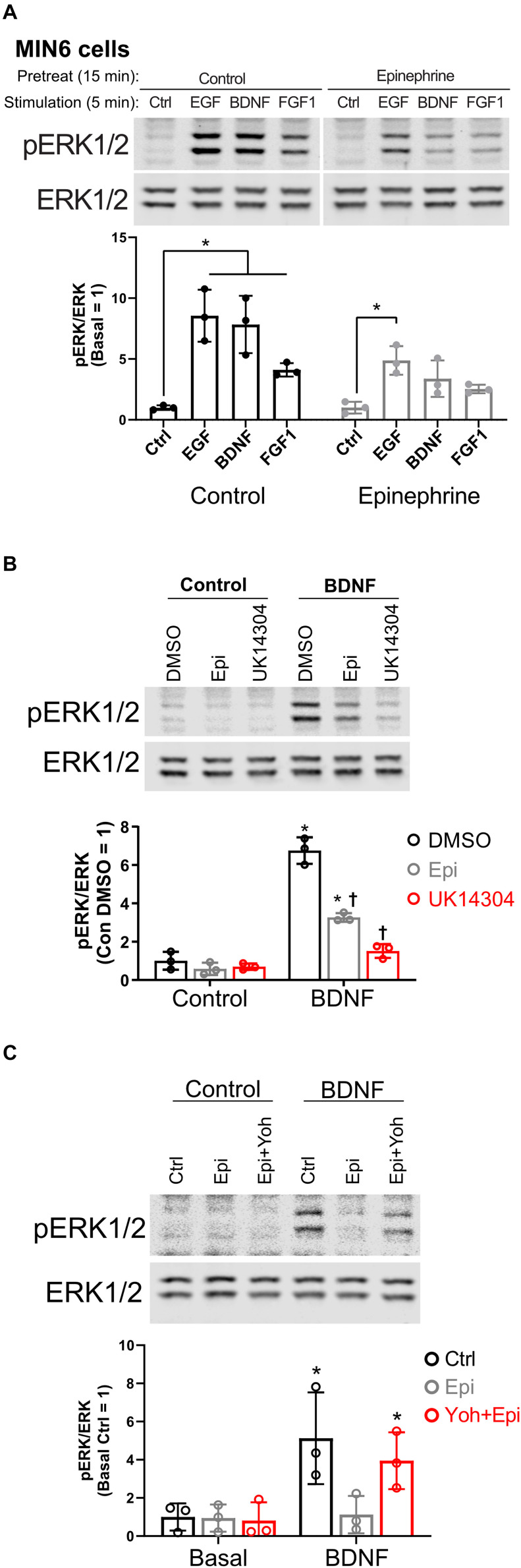
α_2_-adrenergic stimulation suppresses receptor tyrosine kinase signaling in MIN6 β cells. **(A)** To determine the effects of epinephrine pretreatment on receptor tyrosine kinase signaling in β cells, MIN6 cells were preincubated in KRBH with 2 mM glucose for 1 h 45 min before addition of epinephrine (10 μM) for 15 min. Cells were stimulated with the indicated ligand for 5 min (EGF 10 ng/ml; BDNF 10 ng/ml; FGF1 10 ng/ml). Immunoblots are shown for phospho-ERK1/2 (pERK1/2) and total ERK1/2, and data are the mean ± SD for three independent experiments. *P < 0.05 vs Ctrl by two-way ANOVA with Dunnett’s multiple-comparison test. **(B)** To confirm that α_2_-adrenergic stimulation prevents BDNF-stimulated signaling, MIN6 cells were preincubated in KRBH for 1 h 45 min before treatment with 0.1% DMSO, 10 μM epinephrine (Epi), or 10 μM UK14304 for 15 min. Cells were then stimulated with BDNF (10 ng/ml) for 5 min. Immunoblots are shown for phospho-ERK1/2 (pERK1/2) and total ERK1/2, and data are the mean ± SD of three independent experiments. *P < 0.05 for Control vs BDNF and ^†^*P* < 0.05 for DMSO vs drug by 2-way ANOVA with Tukey’s multiple-comparison test. **(C)** To determine if epinephrine-mediated inhibition of BDNF-TrkB signaling is due to its action on the α_2_-adrenergic receptor, MIN6 cells were preincubated in glucose-free KRBH for 1 h and 40 min and then treated with or without the α_2_-adrenergic receptor antagonist yohimbine (10 μM). After 5 min, epinephrine (5 μM) was added as indicated. After 15 min, cells were stimulated with BDNF (10 ng/ml) for 5 min. Immunoblots for pERK1/2 and total ERK1/2 are shown with bar graph quantitation being the mean ± SD of three independent experiments.

In our studies of β cell ERK1/2 activation, we noted an interaction between signaling downstream of RTKs and α_2_-adrenergic receptor stimulation. To expand upon these observations, we stimulated MIN6 β cells with different RTK ligands to examine the effects of epinephrine. EGF, BDNF, and FGF1 stimulated ERK1/2 phosphorylation within 5 min (Figure 1A). Pretreatment with epinephrine for 15 min blocked downstream phosphorylation of ERK1/2 to varying degrees depending on the RTK in question (Figure 1A). We found that EGF signaling to ERK1/2 was partially inhibited by epinephrine (Figure 1A); however, BDNF- and FGF1-induced signaling appeared more sensitive. We chose BDNF-TrkB for our experiments because of its sensitivity to epinephrine and because it is relatively underexplored compared to other RTK signaling pathways in β cells.

To confirm the involvement of that α_2_-adrenergic receptor activation, we tested the isoform-selective adrenergic agonist UK14304, which also suppressed BDNF-TrkB signaling to ERK1/2 in MIN6 cells (Figure 1B). We also found that epinephrine’s effects on BDNF-TrkB signaling were prevented by the α_2_-adrenergic receptor antagonist yohimbine (Figure 1C).

### TrkB Is Expressed in Human Islets and Promotes Cell Growth in MIN6 β Cells

**FIGURE 2:**
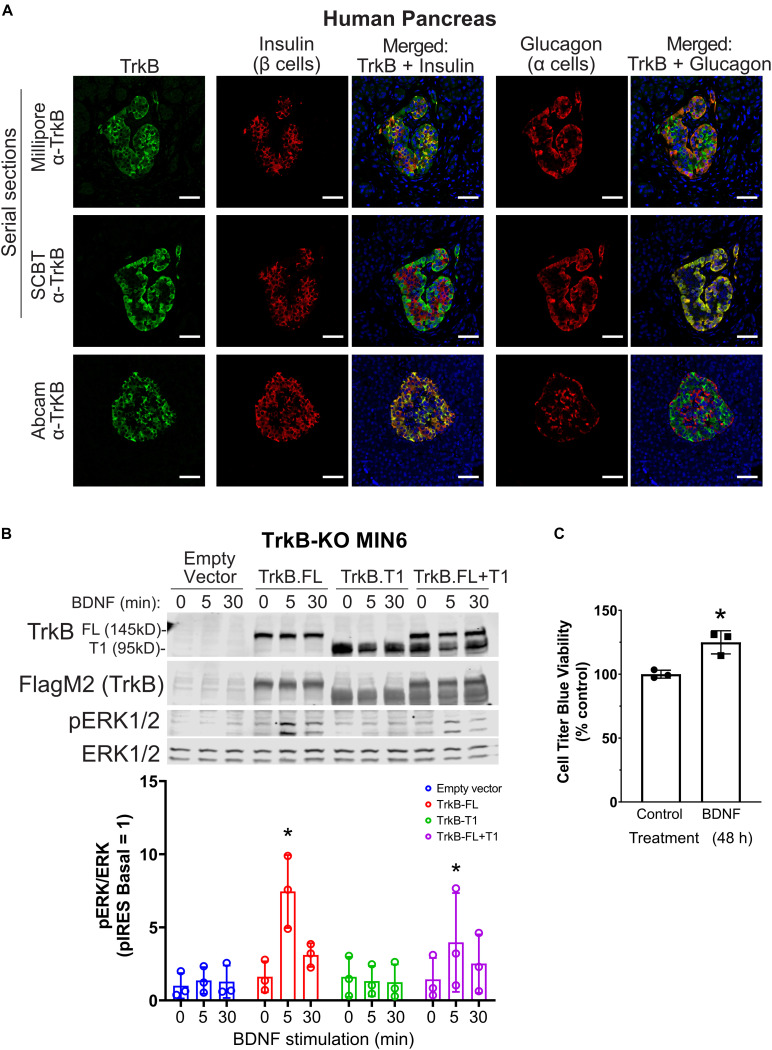
TrkB is expressed in pancreatic islets and chronic BDNF signaling promotes insulin secretion and β cell growth. **(A)** Human pancreas tissue sections were immunostained with antibodies against TrkB (shown in green), insulin to stain β cells (shown in red), glucagon to stain α cells (shown in red), and DAPI to stain nuclei (shown in blue). For the example shown of Millipore and Santa Cruz anti-TrkB antibody staining, serial sections from the same tissue block were stained with different TrkB antibodies and the same islet was located for imaging. Separate panels are shown to illustrate the overlap of TrkB with β cells (insulin) and TrkB with α cells (glucagon). Overlapping regions of green TrkB staining and red insulin/glucagon staining show up yellow. Data are representative of imaging from 2 human pancreas tissue donors. Scale bar, 50 μm. **(B)** To confirm that TrkB-FL is indeed the BDNF receptor signaling to ERK1/2 in β cells, TrkB KO MIN6 cells were transfected with plasmids expressing full-length TrkB (TrkB-FL), TrkB-T1, both, or empty vector (pIRES-3xFlag-dsRed). After 48 h cells were preincubated in KRBH with 4.5 mM glucose for 2 h and stimulated with 10 ng/ml BDNF for 5 and 30 min. Data are the mean ± SD of three independent experiments. *P < 0.05 for 0 vs 5 min of BDNF stimulation by two-way ANOVA using Dunnett’s multiple-comparison test. **(C)** To assess pro-growth effects of chronic BDNF stimulation, MIN6 cells plated in 96-well dishes were incubated for 48 h with BDNF (100 ng/ml) followed by Cell Titer Blue assay for viability. Bar graph is the mean ± SD from three independent passages of cells. *P < 0.05 Control vs. BDNF by Student’s *t*-test.

TrkB was reported to be expressed only in α cells (Shibayama and Koizumi, 1996; Hanyu et al., 2003); however, given our β cell line data, we sought to confirm expression in human islets with multiple antibodies. TrkB was detected in both β and α cells in human (Figure 2A) and mouse (Supplementary Figure 1A) pancreatic islets by immunocytochemistry with independently validated anti-TrkB antibodies (Supplementary Figure 1B). The NTRK2 gene encodes multiple isoforms of TrkB. The major forms are full-length kinase domain-containing TrkB (TrkB.FL) and a truncated form, TrkB.T1, which is missing in the kinase domain (Fenner, 2012). A TrkB antibody against a C-terminal epitope only found in TrkB.FL showed primarily α cell labeling (Figure 2A; SCBT), in agreement with previous work (Shibayama and Koizumi, 1996; Hanyu et al., 2003). However, antibodies with extracellular N-terminal epitopes labeled both α and β cells (Figure 2A; Millipore, Abcam). We found that clonal rodent β cell lines responded to as little as 2.5 ng/ml BDNF, leading to activation of ERK1/2 within 5 min of stimulation (Supplementary Figures 1C–E). BDNF had a negligible effect on the phosphorylation of Akt but increased S6 phosphorylation at 30 min (Supplementary Figure 1F).

BDNF-stimulated activation of ERK1/2 was blocked by small-molecule TrkB inhibitors (GNF-5837 and lestaurtinib) (Supplementary Figures 1G,H) as well as by CRISPR/Cas9-mediated knockout of TrkB (TrkB-KO) (Figure 2B). Multiple clonal lines of TrkB knockout MIN6 cells were confirmed to lack TrkB by immunoblotting and verified to retain glucose-induced ERK1/2 activation (Supplementary Figure 1I). BDNF-stimulated ERK1/2 signaling was rescued upon transient re-expression of TrkB.FL but not TrkB.T1 (Figure 2B). Additionally, we observed that 48 h of BDNF treatment increased viability (Figure 2C). We did not observe any effects of chronic BDNF treatment on glucose-stimulated insulin secretion under similar conditions (Supplementary Figure 1J).

### Epinephrine Inhibits TrkB Signaling at the Receptor Level and Only at Micromolar Concentrations

**FIGURE 3:**
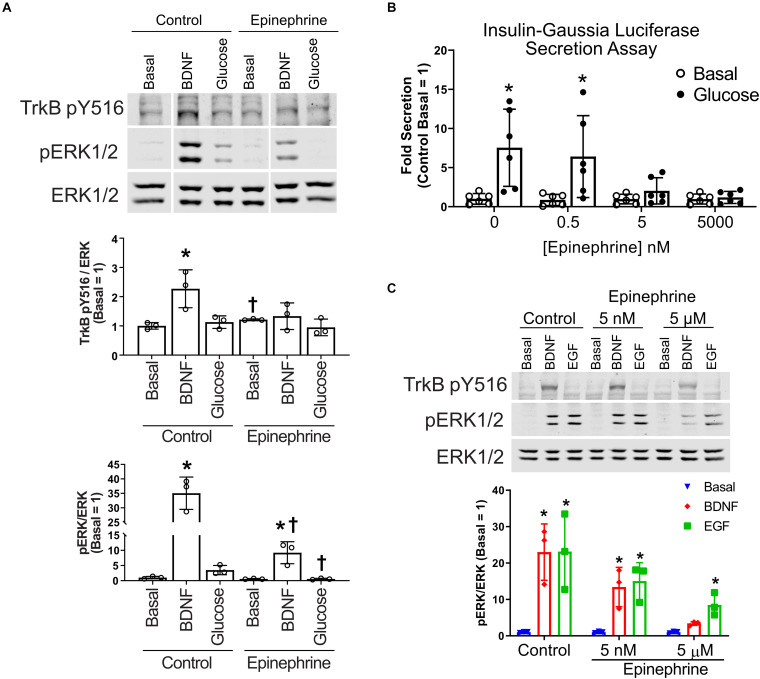
Epinephrine suppresses BDNF signaling to ERK by blocking TrkB activation. **(A)** To determine if epinephrine affected the upstream activation of TrkB itself, MIN6 cells were preincubated in KRBH containing 2 mM glucose for 1 h 45 min before addition of epinephrine (10 μM) for 15 min. Cells were then stimulated with BDNF (10 ng/ml) or glucose (20 mM) for 5 min. Western blot analysis shows phosphorylated TrkB (pY516) and ERK1/2 (pERK1/2) normalized to total ERK1/2 (*N* = 3). Vertical white lines indicate that intervening lanes have been spliced out. *P < 0.05 vs. respective basal. ^†^*P* < 0.05 Control vs. Epinephrine by Student’s *t*-test. **(B)** InsGLuc-MIN6 cells were preincubated in glucose-free KRBH for 1 h followed by stimulation with glucose (20 mM) in the presence or absence of epinephrine (0.5 nM, 5 nM, or 5 μM) for 1 h. Supernatant was collected for Gaussia luciferase assays and the data are reported as the fold change with respect to basal unstimulated cells (*N* = 6). All graphs are the mean ± SD. *P < 0.05 Basal vs. Glucose by two-way ANOVA using Dunnett’s multiple-comparison test. **(C)** MIN6 cells were treated as in panel **(A)**; however, the cells were pretreated with either 5 nM or 5 μM epinephrine prior to stimulation with EGF or BDNF for 5 min. Western blot analysis shows only 5 μM epinephrine suppressed pTrkB (*N* = 2) and pERK1/2 (*N* = 3) signaling by BDNF. Bar graph is the mean ± SD. *P < 0.05 vs. respective Basal by two-way ANOVA using Dunnett’s multiple-comparison test.

To determine how α_2_-adrenergic stimulation could prevent BDNF-TrkB signaling to ERK1/2, we probed the upstream phosphorylation state of TrkB itself. Typically, BDNF stimulates autophosphorylation of the TrkB receptor at several tyrosine residues (Huang and Reichardt, 2003). We found that BDNF-induced tyrosine autophosphorylation of TrkB was blocked by epinephrine in MIN6 β cells (Figure 3A), raising the possibility of direct effects on the TrkB receptor tyrosine kinase. Because nanomolar concentrations of epinephrine are sufficient to inhibit glucose-stimulated insulin secretion in our InsGLuc-MIN6 reporter cells (Figure 3B), we tested the ability of both 5 nM and 5 μM epinephrine to affect MIN6 responses to BDNF or EGF. 5 nM epinephrine suppressed neither BDNF or EGF signaling to ERK nor TrkB tyrosine phosphorylation, while 5 μM epinephrine blocked BDNF signaling, yet EGF retained its significant ability to activate ERK1/2 (Figure 3C).

### Epinephrine-Mediated Inhibition of BDNF-TrkB Signaling Depends on G_i_ but Does Not Involve Calcium Influx or cAMP Generation

**FIGURE 4:**
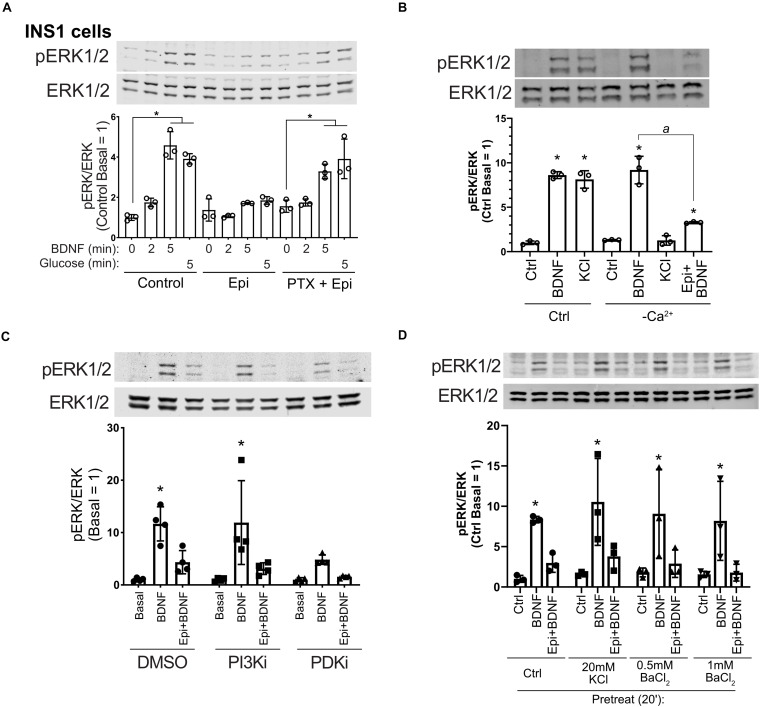
Epinephrine blockade of BDNF signaling depends on Gα_i/o_ but is unaffected by Ca^2+^ influx, PI3K/PDK1 inhibition, or treatment with KCl or BaCl_2_. **(A)** To determine the dependence of epinephrine on G_i/o_ activation, INS1 β cells were treated with 200 ng/ml pertussis toxin (PTX) for 18 h in the culture medium. Cells were then incubated in KRBH with 2 mM glucose in the continued presence or absence of PTX for 2 h. Prior to stimulation, cells were treated with or without 10 μM epinephrine for 15 min. Cells were stimulated with or without 10 ng/ml BDNF or 20 mM glucose for the indicated time. Data are the mean ± SD of three independent experiments. *P < 0.05 vs respective basal by two-way ANOVA using Dunnett’s multiple-comparison test. **(B)** To determine the requirement of Ca^2+^ influx for BDNF-TrkB signaling, MIN6 cells were preincubated in normal (Ca^2+^-containing) or Ca^2+^-free KRBH (compensated with additional 2 mM MgCl_2_) without glucose for 2 h. 15 min prior to stimulation, 10 μM epinephrine was added where indicated. Cells were stimulated with 10 ng/ml BDNF or 50 mM KCl for 5 min. Data are the mean ± SD. *P < 0.05 compared to respective basal; a, *P* < 0.05 by one-way ANOVA using Tukey’s multiple-comparison test. **(C)** To determine if PI3K/PDK1 inhibition prevents the effects of epinephrine on BDNF signaling, MIN6 cells were preincubated for 1.5 h in glucose-free KRBH and then treated with DMSO (0.1%), GDC-0941 (250 nM), or GSK2334470 (250 nM) for 15 min. Cells were then treated or not with 5 μM epinephrine for 15 min before stimulation with BDNF (10 ng/ml) for 5 min. Bar graph represents the mean ± SE for four independent experiments. *P < 0.05 vs. Basal by two-way ANOVA with Dunnett’s multiple-comparison test. **(D)** To block potassium channels potentially involved in membrane hyperpolarization, MIN6 cells were preincubated in glucose-free KRBH for 1 h and 40 min at which point KCl or BaCl_2_ were added. At 1 h and 45 min, epinephrine (5 μM) was added. At 2 h cells were stimulated with BDNF for 5 min and then harvested for Western blot analysis. All data are the mean ± SD of *N* = 3 experiments. *P < 0.05 by two-way ANOVA using Dunnett’s multiple comparisons test.

Epinephrine inhibits insulin secretion in β cells through α_2_-adrenergic receptors linked to Gα_i_ (Straub and Sharp, 2012). To delve further into the mechanism of epinephrine-mediated blockade of BDNF-TrkB signaling, we treated cells with pertussis toxin (PTX) which inactivates Gα_i_. INS1 β cells were used because in our experience they exhibited a more robust response to PTX than MIN6 cells, and INS1 cells responded well to BDNF (Supplementary Figure 1C). PTX prevented the effects of epinephrine on BDNF- and glucose-mediated activation of ERK1/2 (Figure 4A), indicating a requirement for Gα_i/o_ signaling.

Cross talk between receptor tyrosine kinases and GPCRs has been found (Marty and Ye, 2010), although little is known about these pathway interactions in β cells. One mechanism Gα_i/o_ uses to suppress β cell function is through inhibition of adenylyl cyclase (Straub and Sharp, 2012). Therefore, we performed a series of experiments to determine the cross talk between BDNF-TrkB and pathways that generate cAMP. We tested whether BDNF exhibited cross talk with epinephrine, glucagon-like peptide 1 receptor (GLP-1R) agonism, cAMP analogs, and glucose. Pretreatment with GLP-1R agonists GLP-1 or exendin-4, or with the cAMP analog Sp-8Br-cAMPS, enhanced ERK1/2 phosphorylation in response to BDNF in the presence of glucose (Supplementary Figures 2A,B), suggesting interactions among TrkB, the GLP-1 receptor/cAMP, and glucose metabolic pathways. Epinephrine pretreatment dramatically inhibited ERK1/2 activation in response to either glucose, BDNF or their combination.

We also tested whether BDNF on its own can induce cAMP generation in MIN6 cells expressing a bioluminescence-resonance energy transfer-based cAMP reporter (cAMP sensor using YFP-Epac-RLuc or CAMYEL) (Jiang et al., 2007; Guerra et al., 2017). While the known G_s_ activator GLP-1 increased cAMP (Holst, 2007), BDNF had no effect, either alone or in combination with glucose or GLP-1 (Supplementary Figure 2C). However, through inhibition of adenylyl cyclase, Gα_i/o_ suppresses cAMP generation, and basal levels of cAMP may play a role in supporting BDNF-TrkB signaling (Ji et al., 2005). Therefore, we tested whether adenylyl cyclase inhibitors dideoxyadenosine (ddAd) and SQ22536 had the same effect as epinephrine. These compounds did not impact BDNF-stimulated ERK1/2 activation (Supplementary Figure 2D). Therefore, while BDNF-TrkB signaling can synergize with cAMP to enhance ERK1/2 activation, these data suggest against a requirement for cAMP production for BDNF-TrkB signaling in MIN6 β cells.

We also examined the involvement of calcium influx, which regulates ERK1/2 activation in β cells and is inhibited by epinephrine (Straub and Sharp, 2012). Eliminating calcium influx by removal of calcium from the incubation buffer had no impact on BDNF signaling to ERK1/2, nor did it prevent the inhibiting effect of epinephrine on BDNF-induced ERK1/2 activation. KCl-mediated depolarization no longer activated ERK1/2 in the absence of calcium (Figure 4B), demonstrating the mechanistic dichotomy between growth factor and depolarization-stimulated ERK1/2 activation.

### Epinephrine Inhibition of RTKs Is Not Prevented by PI3K/PDK1 Inhibition or by Blockade of Potassium Channels With KCl or BaCl_2_

Inhibition of the phosphoinositide-3-kinase signaling pathway is reported to abrogate the hyperpolarizing effects of micromolar epinephrine (Zhang et al., 2009). We pretreated MIN6 β cells with well-characterized inhibitors for PI3K (GDC-0941) and PDK1 (GSK2334470) followed by epinephrine and analyzed the response to BDNF. These inhibitors did not rescue BDNF-induced ERK1/2 activation (Figure 4C), suggesting that the PI3K/PDK1 pathway is not required for the G_i_-dependent blockade of TrkB activation.

Sieg et al. (2004) found that epinephrine hyperpolarizes cells in a PTX-sensitive manner through unidentified K^+^ channels that can be blocked by low-dose (20 mM) KCl or 0.5–1 mM BaCl_2_. We tested these conditions and found that treating with KCl or BaCl_2_ prior to addition of micromolar epinephrine did not rescue BDNF-TrkB signaling to ERK1/2 (Figure 4D). Another possible explanation is that epinephrine induces TrkB receptor internalization. However, surface biotinylation experiments did not show a significant effect of epinephrine on the amount of surface TrkB (Supplementary Figure 3), suggesting alternative mechanisms.

## Discussion

### How Does α_2_-Adrenergic Stimulation Block Activation of RTKs Like TrkB?

In addition to the events that distinguish signaling by BDNF and glucose, the unexpected sensitivity of BDNF to inhibition by α_2_-adrenergic agonism suggests a connection between signaling by BDNF and insulin secretagogues. RTKs share certain signaling pathways; the Ras-ERK1/2, PI3K-Akt and PLCγ pathways are the most recognized (Minichiello, 2009). Depending on the context, different ligand–receptor family members may signal independently within the same cell to different pathways or exhibit inter-pathway cross talk (Coster et al., 2017), but the exact molecular mechanisms are not always clear. Other inputs such as glucose-stimulated metabolic pathways can act on some of the same signaling pathways seemingly by independent mechanisms (Khoo and Cobb, 1997; Khoo et al., 2004; Kalwat et al., 2013). α_2_-Adrenergic signaling is well-known to antagonize insulin secretion in β cells, largely through heterotrimeric G_i/o_ proteins (Gibson et al., 2006; Zhao et al., 2010; Straub and Sharp, 2012; Ito et al., 2017). In tandem with this effect, α_2_-adrenergic signaling blocks glucose-stimulated ERK1/2 phosphorylation (Gibson et al., 2006), by mechanisms including inhibition of adenylyl cyclase and blockade of calcium influx. We found that BDNF signaling through the ERK1/2 pathway is also blocked in β cells by epinephrine through a G_i_-dependent mechanism at the level of TrkB activation, indicating that essential β cell regulatory inputs are shared between RTKs and glucose-stimulated signaling pathways.

G_i_-dependent cross talk between α_2_-adrenergic receptors and RTKs is a relatively unexplored aspect of β cell signaling. GPCR-RTK cross talk has been observed to activate RTK pathways, but reports of G protein inhibition of RTKs are uncommon (Marty and Ye, 2010). Endogenous plasma epinephrine concentrations in humans normally range from 10 to 100 pg/ml (54.5–545 pM) (Dodt et al., 1997; Kienbaum et al., 1998) but can increase to 1,000 pg/ml (5.4 nM) (Clutter et al., 1980) during infusions of drugs or epinephrine itself. Micromolar and nanomolar concentrations of epinephrine have been proposed to inhibit insulin secretion through different mechanisms (Ito et al., 2017). In the case of nanomolar concentrations of epinephrine, cAMP-TRPM2 channel activity is suppressed, blunting glucose-induced insulin secretion, although sulfonylurea-induced secretion is unaffected (Ito et al., 2017). At ≥1 μM epinephrine, secretion under nearly all conditions is inhibited and the plasma membrane is hyperpolarized. In work from Peterhoff et al., 1 μM epinephrine was shown to inhibit adenylyl cyclase and hyperpolarize the plasma membrane in wild-type but not in α_2__A/C_-adrenergic receptor knockout β cells, suggesting that the actions of micromolar concentrations of epinephrine occur specifically through its receptor (Peterhoff et al., 2003). It is worth noting recent findings showing that even relatively low concentrations of ligand in the aqueous phase above cells can become concentrated at the cell membrane due to interactions with the phospholipid bilayer or receptors (Gherbi et al., 2018), and GPCRs may also be activated by ultralow ligand concentrations (Civciristov et al., 2018).

Because activation of RTK signaling by BDNF in MIN6 β cells appears unaffected by changes in intracellular calcium and did not induce cAMP on its own, suppression of these mechanisms is unlikely to account for the effect of epinephrine on BDNF-TrkB signaling. However, we found that micromolar concentrations of epinephrine were required for its inhibitory activity on TrkB. Why nanomolar epinephrine fails to block BDNF-TrkB signaling when it is also known to activate G_i/o_ under those conditions is an open question. Possible mechanisms may include membrane hyperpolarization or activation of G protein-gated inward rectifier potassium channels (Iwanir and Reuveny, 2008), although it is unclear how membrane hyperpolarization could affect TrkB. One possibility is that G_i/o_ binds directly to TrkB to inhibit its activation, as was shown for G_i/o_ binding to insulin receptor in β cells (Kim et al., 2012); however, mechanisms for the concentration-dependent effects of epinephrine in such a process are unclear.

### What Is the Role for BDNF-TrkB Signaling in β Cells?

BDNF and its receptor TrkB mediate aspects of neuronal development and differentiation and are involved in whole-body energy homeostasis (Hutchison, 2012, 2013) and diabetes (Verge et al., 2014). BDNF can indirectly regulate islet hormones, like glucagon, through actions in the hypothalamus and innervation of the islet (Gotoh et al., 2013) and was suggested to have direct effects on α cells (Hanyu et al., 2003). However, TrkB is expressed in both rodent and human β and α cells, as supported by our data and others (Shibayama and Koizumi, 1996; Hanyu et al., 2003; Uhlen et al., 2015; DiGruccio et al., 2016; Segerstolpe et al., 2016; Fulgenzi et al., 2020). TrkB is expressed as two splice isoforms, full-length TrkB (TrkB.FL) and TrkB.T1 which is missing the cytosolic kinase domain, instead containing a short distinct cytosolic tail (Fenner, 2012). Recent work from Fulgenzi et al. (2020) has demonstrated that the TrkB splice isoform, TrkB.T1, is involved in BDNF-induced insulin secretion and that BDNF can induce insulin secretion from human islets at low glucose concentrations. The relative amounts of TrkB.FL and TrkB.T1 in islet β and α cells has not been defined and could potentially explain the different staining patterns we observe with antibodies to different epitopes. While TrkB.T1 mRNA expression is much greater than full-length TrkB in β cells, TrkB.FL is indeed expressed and even a relatively low amount of RTK at the protein level is sufficient for signaling. We have observed that even >90% knockdown of TrkB protein by siRNA was insufficient to blunt BDNF-stimulated ERK1/2 activation. Not until TrkB protein was eliminated completely by CRISPR/Cas9 did we prevent BDNF-ERK1/2 signaling (Figure 2B).

There are multiple studies linking circulating BDNF concentration to type 2 diabetes in humans and mice (Krabbe et al., 2007; Sha et al., 2007; Li et al., 2016; Murillo Ortiz et al., 2016) as well as in type 1 diabetic patients (Tonoli et al., 2015). Treating db/db mice with BDNF lowered blood glucose, and increased pancreatic insulin content (Tonra et al., 1999) and β cell area and staining intensity were increased (Yamanaka et al., 2006). BDNF may also have a cytoprotective role in the islet because treatment with BDNF prevented RIN5F β cell death in response to alloxan, streptozotocin, doxorubicin, and benzo(a)pyrene (Bathina et al., 2016). Additionally, we observed that the stimulatory concentration of BDNF is well within the range of the circulating hormone (Krabbe et al., 2007; Dell’Osso et al., 2009; Matthews et al., 2009; Karczewska-Kupczewska et al., 2012; Kurita et al., 2012; Pillai et al., 2012). These studies indicate the need for further analysis of the effects and mechanisms of action of BDNF-TrkB signaling in pancreatic islets.

TrkB is known to exhibit cross talk with other kinases, including Src-family kinases (Huang and McNamara, 2010), Ret (Esposito et al., 2008), and the EGF receptor (Puehringer et al., 2013). Oligomerization between receptor kinases TrkA and TrkB could potentially be a contributor to the actions of NGF and may also contribute to BDNF-TrkB signaling not reflected by ERK1/2 activity. Another factor that may complicate interpretation of BDNF function is the expression of isoforms lacking the kinase domain. In our knockout-rescue experiments, TrkB.T1 seemed to suppress the activity of the full-length receptor, as has been suggested in other systems (Eide et al., 1996; Fryer et al., 1997; De Wit et al., 2006). In the future, specific deletion of TrkB.FL from different islet cell types in mice from early in development or in the adult phase may deconvolute roles for TrkB.FL and TrkB.T1 in islet development and function.

### Future Directions

Whether epinephrine or G_i/o_ activation impacts RTK signaling in other cell types is an open question. Adrenergic stimulation of β cells has been suggested to impair β cell growth at near micromolar concentrations (Zhao et al., 2014), and while cAMP is potentially involved, other mechanisms including suppression of RTK signaling may be at work in such conditions.

Future studies are required to place these actions of BDNF in an *in vivo* context to assess their metabolic impact, as well as to elucidate the mechanism underlying the unanticipated finding that epinephrine prevents activation of TrkB itself by BDNF. If any components of that mechanism are pharmacologically targetable, it may be possible to modulate TrkB or other RTK signaling in islets *in vivo* for therapeutic benefit. Notably, single-nucleotide polymorphisms in and near the ADRA2A gene (encoding α_2__A_-adrenergic receptor) have been correlated with increased fasting glycemia and type 2 diabetes risk (Liggett, 2009; Dupuis et al., 2010; Talmud et al., 2011; Langberg et al., 2013); impaired glucose-stimulated insulin secretion is a factor in this increased risk. Our findings suggest there is potential for contribution of altered adrenergic cross talk with RTK signaling in disease. In addition, these results have implications for other systems in which adrenergic and receptor tyrosine kinase signaling may converge, such as cancer (Hui et al., 2008; Powe et al., 2011; Stock et al., 2013).

## Data Availability Statement

The raw data supporting the conclusions of this article will be made available by the authors, without undue reservation.

## Author Contributions

MK: conceptualization and formal analysis. MK, ZH, DB, and KM: investigation. MK and MC: writing—original draft, supervision, and funding acquisition. All authors contributed to the article and approved the submitted version.

## Conflict of Interest

The authors declare that the research was conducted in the absence of any commercial or financial relationships that could be construed as a potential conflict of interest.

## Footnotes

1 http://www.ihcworld.com/_protocols/epitope_retrieval/citrate_buffer.htm
