# Supplementary material for: α2-Adrenergic Disruption of β Cell BDNF-TrkB Receptor Tyrosine Kinase Signaling
Source: Front Cell Dev Biol. 2020 Oct 15;8:576396. doi: 10.3389/fcell.2020.576396 (PMC7593622; doi:10.3389/fcell.2020.576396)
Supplement: Supplementary file 1 [file Data_Sheet_1.pdf]

Supplementary Material

1 Supplementary Methods

1.1 Microscopy of mouse pancreas and NIH-3T3-TrkB cells

Paraffin-embedded pancreas tissue sections from db/db mice were probed for TrkB expression using the Millipore antibody, as with human pancreas tissue sections. We tested TrkB antibodies using NIH-3T3-TrkB cells (Soppet et al. 1991) by immunofluorescence microscopy. We determined that antibodies from Millipore and Santa Cruz Biotechnologies detected human TrkB. Two other antibodies from Santa Cruz Biotechnology and Origene were not optimal. In other immunoblotting experiments we observed that rabbit anti-TrkB from Abcam (Cat# ab134155, 1:1000) also detected TrkB.FL in differentiated SHSY5Y cells.

1.2 Intracellular cAMP measurements

We used the Epac-based bioluminescence resonance energy transfer (BRET) sensor for cAMP (CAMYEL) in MIN6 cells as previously described (Jiang et al. 2007, Guerra et al. 2017). Briefly, BRET assays were performed on the Synergy H1 microplate reader (BioTek). MIN6 CAMYEL cells were preincubated in KRBH without glucose for 1.5 h followed by addition of 2.55 μM coelenterazine-h for 15 min. Assays were performed in triplicate. Emission signals at 485/20 and 528/20 nm (center/bandpass) were measured every 0.8 s during a 3 min baseline measurement. Stimuli were then added and cells were measured for an additional 10 min.

1.3 Surface Biotinylation Assay

Sulfo-NHS-SS-biotin was resuspended in DMSO and stored in single-use 400X (200 mg/mL) aliquots at -20°C. MIN6 cells were preincubated in glucose-free KRBH without BSA for 1.75 h prior to treatment with or without 10 µM epinephrine for 15 min. KRBH was removed, cells placed cold shaker in 4°C walk-in fridge and cold PBS was added containing with Sulfo-NHS-SS-biotin (0.5 mg/ml) for 30 min to label surface exposed proteins. 3 x 10 min washes with 0.1M glycine to stop the reaction. Cells were lysed in lysis buffer (2% NP-40, 150 mM NaCl, 0.01% SDS, 50 mM HEPES pH 7.5 and protease/phosphatase inhibitors) and 2 mg cleared lysates were used in streptavidin-agarose pulldowns. The precipitated proteins were run on SDS-PAGE and immunoblotted for TrkB. ATP1A1 and ADRA2A were also blotted as controls. Surface TrkB and ADRA2A were normalized to surface ATP1A1.

1.4 Insulin measurements

Prior to stimulation, MIN6 cells were washed twice with and incubated for 2 h in freshly prepared glucose-free modified Krebs-Ringer bicarbonate buffer (MKRBB: 5 mM KCl, 120 mM NaCl, 15 mM HEPES, pH 7.4, 24 mM $NaHCO_3$, 1 mM $MgCl_2$, 2 mM $CaCl_2$, and 1 mg/ml radioimmunoassay-grade BSA). For insulin secretion measurements, supernatants were collected, cleared and stored at -20°C. Secreted and total insulin content were measured using a mouse Insulin ELISA (Mercodia).

1.5 INS-1 cell culture

Rat INS-1 cells were cultured in RPMI-1640 containing 11 mM glucose, 10% FBS, 1 mM sodium pyruvate, 50 μM β-mercaptoethanol, 10 mM Hepes pH 7.4, penicillin/streptomycin as above.

Conditions for pertussis toxin (PTX, Fisher PHZ1174) treatments have been described previously (Taussig et al. 1992, Gibson et al. 2006). Briefly, INS1 cells in 12-well culture dishes were incubated for 24 h in the presence or absence of 200 ng/ml PTX, followed by a 2 h incubation in KRBH with 2 mM glucose also in the presence or absence of 200 ng/ml PTX. Cells were then pretreated with 10 μM epinephrine for 15 min and stimulated with 10 ng/ml BDNF or 20 mM glucose for the indicated times and harvested for immunoblotting.

1.6 Plasmid generation and CRISPR knockout clone generation

Mouse TrkB.FL and TrkB.T1 cDNAs were cloned from MIN6 cell cDNA libraries, generated using the Protoscript II First Strand cDNA Synthesis kit (NEB Cat# E6560S). cDNAs were inserted into pIRES-3xFlag-dsRed by restriction digestion and ligation. pX330-U6-Chimeric_BB-CBh-hSpCas9 was a gift from Feng Zhang (Addgene plasmid #42230) (Cong et al. 2013). Guide RNAs were designed targeting mouse NTRK2 exon 1 and cloned into pX330 following the Zhang lab protocol. The 20 bp guide sequences (#1: 5'-GACCCGCCATGGCGCGGCTC-3', #2: 5'-GGAACCTAACAGCGTTGACC-3') were generated using the CRISPR design tool (http://crispr.mit.edu). Oligonucleotides for cloning the gRNAs are shown in **Table S1**. The oligonucleotides were phosphorylated with T4 PNK and annealed for insertion into the BbsI site of pX330. All plasmids were verified by DNA sequencing (Genewiz).

To generate MIN6 TrkB knockout lines, 35 mm wells of MIN6 cells were co-transfected with 2.5 µg of pX330 containing gRNAs and 1 µg of pEGFP-C2 (Clontech). After 48 h, cells were trypsinized and GFP positive cells were sorted one per well in round bottom 96-well trays by fluorescence activated cell sorting for clonal expansion. After 3-4 of expansion, colonies were trypsinized and seeded into 24-well plates and screened for TrkB protein expression by immunoblotting. Clones were selected which exhibited normal morphology and low basal phosphorylated ERK1/2.

2 Supplementary Figures

Supplementary Figure 1. TrkB is expressed in pancreatic islets and BDNF stimulates the canonical TrkB RTK signaling pathway in β cells.

A) db/db mouse pancreas tissue sections were immunostained for TrkB, insulin, and glucagon. Scale bar, 20 μm.

B) Anti-TrkB immunofluorescent staining in 3T3-TrkB cells to validate antibody suitability.

C) INS1 cells were preincubated 2 h in glucose-free KRBH and then stimulated with glucose (20 mM) or BDNF (10 ng/ml) for 5 min. Data are the mean ± SD. *, $P<0.05$ by Student's t-test.

D,E) MIN6 cells were preincubated in glucose-free KRBH and then treated with increasing doses of BDNF for 5 min. pERK1/2 and ERK1/2 were immunoblotted and quantitation is show below as bar graphs. Data are the mean ± SD of three independent passages of cells. *, $P<0.05$ vs basal by Student's t-test.

F) MIN6 cells were preincubated in KRBH with 2 mM glucose for 2h and then treated with or without 10 ng/ml BDNF for 5 and 30 min. Data are the mean ± SE of four independent experiments. *, P<0.05 by Student's t-test.

G,H) MIN6 cells were preincubated in KRBH with 2 mM glucose for 2 h prior to a 10 min pretreatment with either GNF-5837 (10 µM) or lestaurtinib (Lest, 2 µM) prior to stimulation with BDNF (50 ng/ml). Data are the mean ± SD of three independent experiments. Vertical white lines indicate that intervening lanes have been spliced out. *, P<0.05 by Student's t-test.

I) One wild type and two distinct clonal lines of TrkB KO (KO1, KO2) MIN6 cells were preincubated in KRBH without glucose for 2 h and stimulated with 20 mM glucose for 5 min. Vertical white lines indicate that intervening lanes have been spliced out.

J) MIN6 cells were incubated with BDNF (100 ng/ml) for 24 h followed by incubation in KRBH to assay for glucose-stimulated insulin secretion. Insulin secretion (as % content) was normalized to basal secretion. Bar graph is the mean ± SD from three independent passages of cells. *, P<0.05 for 0 vs 30 min by two-way ANOVA using Dunnett's multiple comparisons test.

Supplementary Figure 2. BDNF-TrkB exhibits crosstalk with GLP-1 and adrenergic signaling pathways in the β cell, but does not induce cAMP.

A) BDNF-TrkB may exhibit crosstalk with cAMP-generating signaling pathways, such as the incretin pathway. To assess this, MIN6 cells were preincubated in glucose-free KRBH for 2 h with or without the addition of 10 ng/ml BDNF (150 min final treatment). Prior to the glucose addition cells were treated with or without 50 nM GLP-1 or 5 µM epinephrine for 15 min. After 2 h, 20 mM glucose was added either alone, to the BDNF pre-treated cells, or in combination with BDNF for 30 min. Phosphorylated ERK1/2 (pERK1/2) and total ERK1/2 immunoblots are shown and the quantitated ratio of pERK/ERK is shown below. Bar graph is the mean ± SD of three independent experiments. *, P<0.05 by two-way ANOVA with Dunnett's multiple comparisons test.

B) To further test the extent to which cAMP may interact with BDNF-TrkB signaling, MIN6 cells were preincubated in glucose-free KRBH for 1 h 45 min before a 15 min pretreatment with the cAMP analog Sp-8Br-cAMPS (50 µM) alongside the GLP1R agonist Exendin-4 (30 nM) and epinephrine (5 µM) as controls. The cells were then stimulated with BDNF (10 ng/ml), with or without glucose (20 mM) for 30 min and harvested for immunoblotting. Both Sp-8Br-cAMPS and Exendin-4 amplified the amount of BDNF-induced ERK1/2 phosphorylation only in the presence of glucose. Data are the mean ± SD of three independent experiments. *,P<0.05 vs Control Basal, †, P<0.05 vs BDNF, ‡, P<0.05 between indicated bars by two-way ANOVA with Tukey's multiple comparisons test.

C) Intracellular cAMP generation was measured using MIN6 CAMYEL cells. Cells were preincubated in KRBH without glucose for 1.5 h and treated with 20 µM coelenterazine for 20 min. After a 3 min baseline measurement, stimuli were injected and BRET signal was monitored. Stimuli concentrations were: 20 mM Glucose, 30 nM GLP-1, 10 ng/ml BDNF. Data are the mean ± SE of three experiments performed in triplicate.

D) To determine if BDNF-TrkB signaling requires cAMP generation by adenylyl cyclase, MIN6 cells were preincubated in glucose-free KRBH for 1 h and 50 min prior to treatment. 10 min pretreatment of MIN6 cells with adenylyl cyclase inhibitors dideoxyadenosine (ddAd, 100 µM) or SQ22536 (100

μM) did not prevent BDNF (10 ng/mL) from stimulating ERK1/2 activation within 5 min, while epinephrine (5 μM) blocked stimulation. *, $P<0.05$ for Basal vs. BDNF by two-way ANOVA with Sidak's multiple comparisons test.

Supplementary Figure 3. TrkB internalization was unaffected by epinephrine.

MIN6 cells were preincubated in KRBH without BSA for 2 h and then stimulated with epinephrine (10 μM) for 15 min. Immediately after, cells were transferred to ice and labeled with NHS-SS-biotin to biotinylate surface proteins, the reaction was quenched with glycine and lysates were prepared for streptavidin bead precipitation and Western blot analysis. The α_2-adrenergic receptor and TrkB bands were normalized to Na/K-ATPase (ATP1A1) and to the basal unstimulated condition. Bar graph is mean ± SD of at least three independent experiments.

3 Supplementary References

Cong, L., F. A. Ran, D. Cox, S. Lin, R. Barretto, N. Habib, P. D. Hsu, X. Wu, W. Jiang, L. A. Marraffini and F. Zhang (2013). "Multiplex genome engineering using CRISPR/Cas systems." Science **339**(6121): 819-823.

Gibson, T. B., M. C. Lawrence, C. J. Gibson, C. A. Vanderbilt, K. McGlynn, D. Arnette, W. Chen, J. Collins, B. Naziruddin, M. F. Levy, B. E. Ehrlich and M. H. Cobb (2006). "Inhibition of glucose-stimulated activation of extracellular signal-regulated protein kinases 1 and 2 by epinephrine in pancreatic beta-cells." Diabetes **55**(4): 1066-1073.

Guerra, M. L., M. A. Kalwat, K. McGlynn and M. H. Cobb (2017). "Sucralose activates an ERK1/2-ribosomal protein S6 signaling axis." FEBS Open Bio **7**(2): 174-186.

Jiang, L. I., J. Collins, R. Davis, K. M. Lin, D. DeCamp, T. Roach, R. Hsueh, R. A. Rebres, E. M. Ross, R. Taussig, I. Fraser and P. C. Sternweis (2007). "Use of a cAMP BRET sensor to characterize a novel regulation of cAMP by the sphingosine 1-phosphate/G13 pathway." J Biol Chem **282**(14): 10576-10584.

Soppet, D., E. Escandon, J. Maragos, D. S. Middlemas, S. W. Raid, J. Blair, L. E. Burton, B. R. Stanton, D. R. Kaplan, T. Hunter, K. Nikolics and L. F. Parade (1991). "The neurotrophic factors brain-derived neurotrophic factor and neurotrophin-3 are ligands for the trkB tyrosine kinase receptor." Cell **65**(5): 895-903.

Taussig, R., S. Sanchez, M. Rifo, A. G. Gilman and F. Belardetti (1992). "Inhibition of the omega-conotoxin-sensitive calcium current by distinct G proteins." Neuron **8**(4): 799-809.

4 Supplementary Table

Supplementary Table 1. All antibody information including RRIDs are provided. Antibodies for immunoblotting were diluted in Licor Blocking buffer (diluted 1:5 in TBS-T). Primary incubation was overnight. Secondary incubation was for 1h. Blots were washed 3 times for 10min each after primary and secondary. For immunofluorescence all blocking was 1 h at RT, primary overnight at 4°C and secondary 1h RT. All washing was 3 times for 10min each in PBS-T at RT. IB, immunoblotting; IF, immunofluorescence. All specialized reagents, primers, and tissue donor information is also provided.

Antibody Details

Antigen	Host	Mol Weight (kDa)	Company	Cat#	RRID	Dilution for IB	Dilution for IF	Epitope
pERK1/2	Mouse	42,44	Cell Signaling Technologies	9106	AB_331768	1:1000	-	
ERK1/2	Rabbit	42,44	In-house	Y691		1:3000	-	
TrkB	Rabbit	145, 95	Millipore	07-225	AB_310445	1:1000	1:100	extracellular domain of rat TrkB
TrkB	Rabbit	145, 95	Abcam	ab134155	AB_2857962	-	1:100	Residues 130-160 in human TrkB
TrkB	Rabbit	145, 95	Santa Cruz Biotechnologies	sc-12	AB_632557	-	1:100	Residues 760-810 of mouse TrkB
TrkB	Mouse	145, 95	Origene	TA500386	AB_2155134	-	1:100	
TrkB	Mouse	145, 95	Santa Cruz Biotechnologies	sc-377218	AB_2801499		1:100	
pY490/516 TrkA/B	Rabbit	145	Cell Signaling Technologies	4619	AB_10235585	1:1000	-	
Insulin	Goat	6	Santa Cruz Biotechnologies	sc-7839	AB_2296108	-	1:300	
Glucagon	Mouse	3.4	Sigma	G2654	AB_259852	-	1:1000	
Akt	Mouse	60	Cell Signaling Technologies	2967	AB_331160	1:1000	-	
pS473 Akt	Rabbit	60	Cell Signaling Technologies	4060	AB_2315049	1:1000	-	
S6 (pS235/244)	Rabbit	32	Cell Signaling Technologies	2211	AB_331679	1:1000	-	
S6	Mouse	32	Santa Cruz Biotechnologies	sc-74459	AB_1129205	1:3000	-	
ATP1A1	Mouse	112	Invitrogen	MA1-16731	AB_2060993	1:1000	-	
ADRA2A	Rabbit	51	Proteintech	14266-1-AP	AB_2636822	1:1000	-	

Secondary Antibodies (IB)

anti-rabbit 680 (red)	Donkey		Licor	92668073	AB_10954442	1:10,000	-	
anti-mouse 800 (green)	Donkey		Licor	92632212	AB_621847	1:10,000	-	

Secondary Antibodies (IF)

anti-rabbit 647	Donkey		Invitrogen	A-31573	AB_2536183	-	1:400	

anti-goat 555	Donkey		Invitrogen	A-21432	AB_141788	-	1:400	
anti-mouse 488	Donkey		Invitrogen	A-21202	AB_141607	-	1:400	

Reagents and other materials

**Name**	**Company**	**Catalog #**
GLP-1	Sigma	G8147
Exendin 4	Fisher	AAJ66726EXD
Lipofectamine 2000	Fisher	11668019
UK14304	Sigma	U104
Yohimbine	Sigma	Y3124
Forskolin	Sigma	F3917
Sp-8Br-cAMPs	Enzo	ALX480025M001
EGF	Gibco	PHG0311
FGF1a	Biolegend	750902
BDNF	Millipore	GF029
Pertussis Toxin (PTX)	Fisher	PHZ1174
Epinephrine	Sigma	E4375
GSK2334470	Apexbio	B2174
GDC-0941	Apexbio	A8210
$BaCl_2$	Sigma	202738
Cell Titer Blue	Promega	G8080
Round-bottom 96 well culture dishes	Corning	3799
Mouse Insulin ELISA	Mercodia	10-1247-10
Sulfo-NHS-SS-biotin	Fisher	PI21331
coelenterazine-h	Fisher	PR-S2011

Primers

**Name**	**Sequence (5' -> 3')**
NTRK2-#1_sense	CACCGACCCGCCATGGCGCGGCTC

NTRK2-#1_antisense	AAACGAGCCGCGCCATGGCGGGTC
NTRK2-#2_sense	CACCGGAACCTAACAGCGTTGACC
NTRK2-#2_antisense	AAACGGTCAACGCTGTTAGGTTCC

Human Pancreatic Donor Tissue Information

UTSW ID#	Diagnosis
18037	Mucinous Carcinoma of the panc (tail)
3394	Intraductal papillary mucinous neoplasm
