# Supplementary figures and images for: α2-Adrenergic Disruption of β Cell BDNF-TrkB Receptor Tyrosine Kinase Signaling
Source: Front Cell Dev Biol. 2020 Oct 15;8:576396. doi: 10.3389/fcell.2020.576396 (PMC7593622; doi:10.3389/fcell.2020.576396)

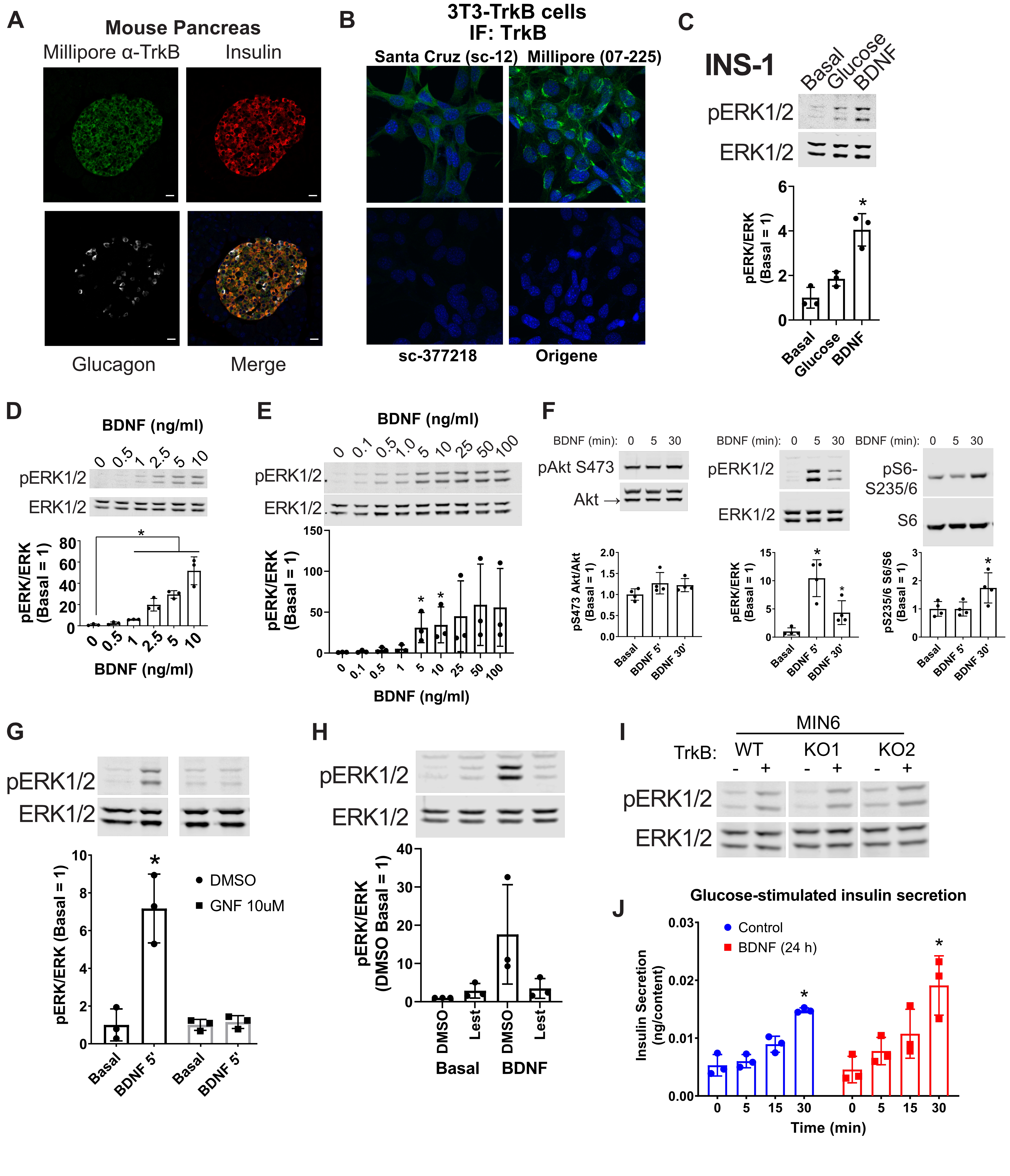

Supplement: Supplementary file 2 [file Image_1.TIFF]

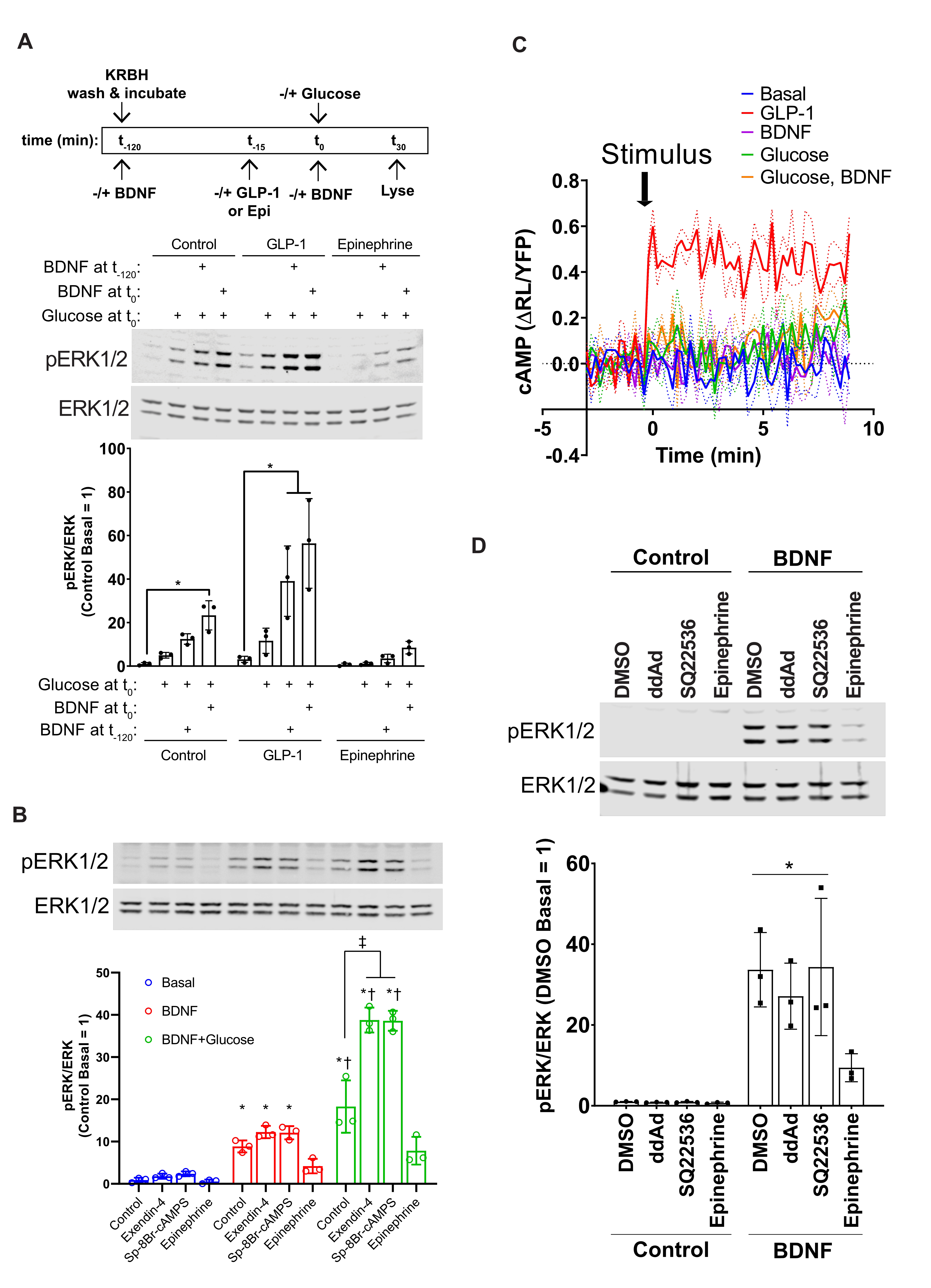

Supplement: Supplementary file 3 [file Image_2.TIFF]

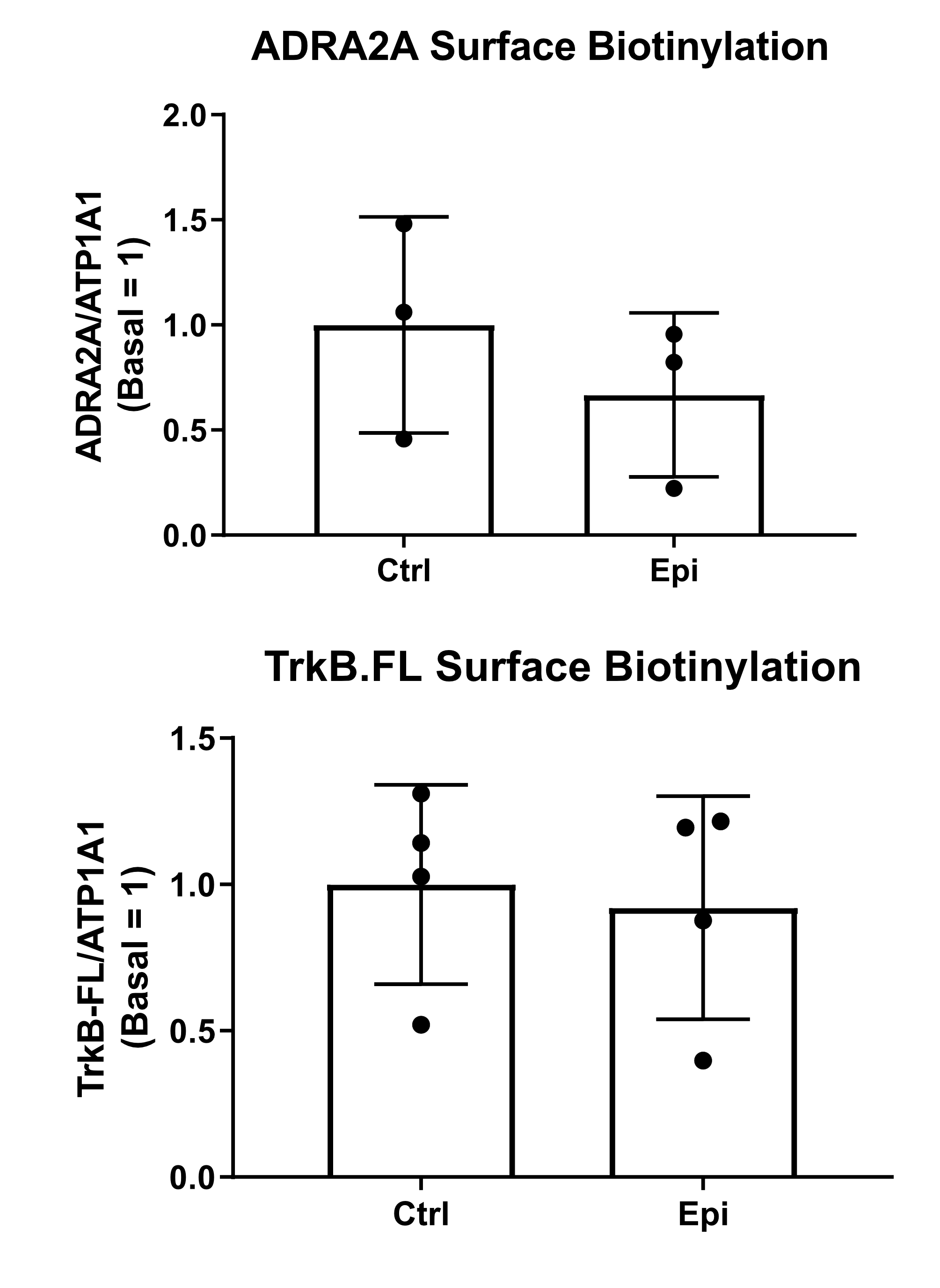

Supplement: Supplementary file 4 [file Image_3.TIFF]
